# Enhanced Antifungal and Wound Healing Efficacy of Statistically Optimized, Physicochemically Evaluated Econazole-Triamcinolone Loaded Silica Nanoparticles

**DOI:** 10.3389/fchem.2022.836678

**Published:** 2022-05-03

**Authors:** Safirah Maheen, Hina Younis, Hafeez Ullah Khan, Syed Salman shafqat, Sajed Ali, Atta Ur Rehman, Saliha Ilyas, Muhammad Nadeem Zafar, Syed Rizwan Shafqat, Abul Kalam, Ahmed A. Al-Ghamdi

**Affiliations:** ^1^ Department of Pharmaceutics, College of Pharmacy, University of Sargodha, Sargodha, Pakistan; ^2^ Department of Chemistry, Division of Science and Technology, University of Education, Lahore, Pakistan; ^3^ Department of Biotechnology, University of Management and Technology Sialkot Campus, Sialkot, Pakistan; ^4^ Department of Pharmacy, Forman Christian College (A Charted University), Lahore, Pakistan; ^5^ Department of Chemistry, University of Gujrat, Gujrat, Pakistan; ^6^ Department of Chemistry, Universiti Malaysia Sarwak, Kota Samarahan, Malaysia; ^7^ Research Center for Advanced Materials Science (RCAMS), King Khalid University, Abha, Saudi Arabia; ^8^ Department of Chemistry, College of Science, King Khalid University, Abha, Saudi Arabia; ^9^ Department of Physics, Faculty of Science, King Abdulaziz University, Jeddah, Saudi Arabia

**Keywords:** econazole, histopathology, *in vitro*-*in vivo* antifungal, mesoporous silica nanoparticles, wound healing, triamcinolone

## Abstract

Co-encapsulated econazole nitrate-triamcinolone acetonide loaded biocompatible, physically stable, and non-irritating mesoporous silica nanoparticles (EN-TA–loaded MSNs) were prepared and optimized by using a central composite rotatable design (CCRD) for providing better therapeutic efficacy against commonly prevailed resistant fungal infections. These drugs loaded MSNs can significantly overcome the deficiencies and problems like short duration of action, requirement of frequent administration, erythema, and burning sensation and irritation associated with conventional drug delivery systems. The stability of optimized drugs loaded MSNs prepared with 100 gm of oil at pH 5.6 with a stirring time of 2 h was confirmed from a zeta potential value of −25 mV. The remarkable compatibility of formulation ingredients was depicted by X-ray diffraction (XRD), differential scanning calorimetry (DSC), and Fourier transform infrared spectroscopy (FTIR) spectra while scanning electron microscopy (SEM) and size analysis represented a very fine size distribution of nanoparticles ranging from 450–600 nm. The CCRD clearly predicted that the optimized parameters of drugs loaded MSNs have better values of percentage yield (85%), EN release (68%), and TA release (70%). Compared to pure drugs, the decreased cytotoxicity of EN-TA–loaded MSNs was quite evident because they showed a cell survival rate of 90%, while in the case of pure drugs, the survival rate was 85%. During *in vivo* antifungal testing against *Candida albicans* performed on three different groups, each consisting of six rabbits, the EN-TA–loaded MSNs were relatively superior in eradicating the fungal infection as a single animal exhibited a positive culture test. Rapid recovery of fungal infection and a better therapeutic effect of EN-TA–loaded MSN were quite evident in wound healing and histopathology studies. Likewise, on the 14th day, a larger inhibitory zone was measured for optimized nanoparticles (15.90 mm) compared to the suspension of pure drugs (13.90 mm). In skin irritation studies, MSNs did not show a grade of erythema compared to pure drugs, which showed a four-fold grade of erythema. As a result, MSNs loaded with combination therapy seem to have the potential of improving patient compliance and tolerability by providing enhanced synergistic antifungal effectiveness at a reduced dose with accelerated wound healing and reduced toxicity of therapeutics.

## 1 Introduction

The most common and clinically significant fungal infections targeting human hair, nails, mucosa, epidermis, and visceral organs are universally distributed (found in animals, soil, and humans), transmitted from person to person, and are life-threatening. Several of these infections are therapeutically resistant (He et al., 2011; Han et al., 2012). Conventional topical dosage forms such as creams, ointments, and pastes are less acceptable owing to side effects such as erythema, burning sensation, rashes, tenderness, and irritation (Ghadiri et al., 2012). They also require high concentrations of antifungal agents such as fluconazole, voriconazole, and itraconazole (He et al., 2011) to enhance effectiveness in severe dermal infections and are difficult to use because of their short duration of action and high viscosity (Agrawal et al., 2010; He et al., 2011). Moreover, the emergence of resistant infections requires treatment with a combination of drugs (Fotouhi et al., 2009). It was intended to develop a formulation to provide long-lasting benefits of an antimycotic, particularly azoles such as econazole nitrate (Mangas-Sánchez et al., 2011) and a corticosteroid such as triamcinolone acetonide, used to treat multiple skin diseases but their use is limited used due to their poor aqueous solubility, poor penetration, and skin itching that eventually limits compliance with patient guidelines (Agrawal et al., 2010; Hu et al., 2015).

Nowadays, drug-loaded mesoporous silica nanoparticles (MSNs) are produced owing to their known safe and biodegradable nature in order to overcome all deficiencies and problems of conventional methods of treatment (Granero and Amidon, 2006). MSNs are considered as a promising pharmaceutical vehicle due to their characteristic mesoporous structure, high specific surface area, high pore volume, high stress resistance, chemical stability, favorable biocompatibility, and surface functionality (Weber et al., 2000; Pradeep et al., 2010) that ensures maximum drug loading, controlled release of different drug molecules, and prolonged therapeutic action, thereby overcoming the problems of conventional systems (Pradeep et al., 2010). The MSNs can support well the administration of medications and promote the healing of wounds such as burn wounds or infectious wounds by promoting re-epithelialization through epidermal maturation and neovascularization (Zwiorek et al., 2004). The current research aims at the formulation of MSNs as an alternative drug delivery system for co-loading econazole nitrate (EN) and triamcinolone acetonide (TA) in order to achieve an effective and rapid healing process and overcome the hydrolysis, drug leakage (Katiyar et al., 2016) and aggregation, toxicity (Draize, 1944), and oxidation problems presented by other novel drug delivery systems, like surfactant-based niosomes (Kiehlbauch et al., 2000), lipid-based liposomes (Gupta and Vyas, 2012) and polymeric micro or nanoparticles (Prajapati et al., 2011).

The studies presented were aimed at optimizing the MSNs produced by a sol-gel process using response surface methodology. The formulation factors such as pH, stirring time, and oil concentration were optimized and their mathematical influence on the percentage yield (PY) and the release of the active ingredients from EN-TA–loaded MSNs was observed. The drug’s loaded nanoparticles were also evaluated for morphology, size, and zeta potential. The EN-TA compatibility with MSNs was evaluated by thermogravimetric analysis (TGA), differential scanning calorimetry (DSC), X-ray diffraction (XRD), and Fourier transform infrared spectroscopy (FTIR). The finally optimized MSNs were then subjected to be analyzed for antifungal studies, cytotoxicity, skin irritation, histopathology, and wound healing studies.

## 2 Materials and Methodology

### 2.1 Chemicals

Econazole nitrate (EN) and triamcinolone acetonide (TA) were provided as gift samples from Harman Pharmaceutics, Lahore, Pakistan, and from Mass Pharma, Lahore, Pakistan, respectively. Hydrochloric acid, absolute ethanol, and ammonium hydroxide were purchased from Sigma Aldrich. Tetraethoxyorthosilicate (TEOS) and vegetable oil were purchased from Uni Chem, Lahore, and Dalda Foods (pvt) Ltd., Pakistan.

### 2.2 Optimization Design

To optimize the formulation variables, traditional methods allow the change of a single factor while all other factors are kept at the same level. In addition, these traditional techniques are unable to operate the simultaneous interactive effect of various factors and require the consumption of a lot of time and auxiliary materials. Therefore, in such situations, the statistical optimization method such as central composite rotatable design (CCRD) appears to be a promising technique for numerically studying the concurrent influence of variables independent experimental parameters on nanoparticles without loss of material and time, and it can be applied in a better way by Design Expert (version 8.0.6.1 Stat-ease, Inc., United States). Before applying the design, the preliminary studies were performed by varying the concentration of all formulation variables. The concentration of TEOS and both drugs had not significantly affected the nanoparticles’ yield and drugs release, so their concentration was initially calibrated and maintained constant (300 mg) throughout the study. In the current work, a five-level three-factor CCRD design was established to statistically study the effects of other formulation factors such as oil concentration (X_1_), stirring time (X_2_), and pH (X_3_) on nanoparticle responses such as percentage yield (Y_1_), EN release (Y_2_), and TA release (Y_3_). The present study used the Design Expert which proposed a total of 20 experimental MSNs formulations (Nandy and Mazumder, 2014).

### 2.3 Synthesis of EN-TA-loaded Mesoporous Silica Nanoparticles

The drug-loaded MSNs (EN-TA-MSNs) were prepared by a sol-gel method. Freshly prepared 0.1 M HCl solution and distilled water were mixed well with ethyl silicate in a beaker at room temperature at 200 rpm. On the other hand, separate solutions of 3% TA and 10% EN in ethanol were prepared. These solutions of drugs were then poured into previously prepared sol with continuous homogenization. The obtained sol containing drugs was cooled to 4 °C, followed by the dropwise addition of 0.08 M NH_4_OH to adjust pH to 5.8. The time required for conversion of sol to gel was recorded (Radin et al., 2009). The prepared gel was directly added to 100 ml of vegetable oil in a dropwise manner with continuous stirring at 1,000 rpm by using a yellow line homogenizer. The stirring process continued until the precipitation of nanoparticles at the bottom of the beaker. The drug-loaded silica nanoparticles were filtered, washed with distilled water, and dried at room temperature. Twenty different nanoparticle formulations were synthesized by varying the levels of formulation factors as proposed by the applied design (Table 1).

**TABLE 1:** Composition of various formulations designed by CCRD and results of percentage yield and drugs release.

Formulation	Formulation variables			Results of responses		
	Vegetable oil conc. (ml)	Stirring time (hours)	pH	Percentage yield (%)	EN release (%)	TA release (%)
F1	65	2.00	4.47	48 ± 1.37	30 ± 3.56	35 ± 4.61
F2	65	2.00	7.00	55 ± 1.91	50 ± 2.43	54 ± 2.86
F3	30	3.00	5.50	38 ± 0.45	38 ± 1.45	42 ± 4.89
F4	65	3.00	7.00	65 ± 3.74	52 ± 5.46	56 ± 2.34
F5	100	3.00	5.50	70 ± 1.23	40 ± 3.79	43 ± 2.87
F6	65	2.00	7.00	55 ± 1.53	50 ± 1.66	54 ± 3.76
F7	65	1.00	7.00	45 ± 0.45	48 ± 5.98	51 ± 2.34
F8	98	2.00	7.00	68 ± 3.61	50 ± 3.95	54 ± 3.66
F9	65	2.00	7.00	55 ± 1.98	50 ± 1.95	54 ± 3.33
F10	65	2.00	7.00	55 ± 0.45	50 ± 3.98	54 ± 1.84
F11	100	3.00	8.50	68 ± 3.54	74 ± 1.45	84 ± 2.36
F12	65	2.00	7.00	55 ± 1.21	50 ± 5.92	54 ± 1.92
F13	100	2.00	8.50	75 ± 0.45	75 ± 1.45	79 ± 1.93
F14	30	3.00	8.50	77 ± 1.52	74 ± 3.87	78 ± 4.67
F15	30	1.00	5.50	40 ± 3.66	37 ± 4.12	43 ± 1.37
F16	65	2.00	7.00	55 ± 1.21	50 ± 5.88	54 ± 3.27
F17	65	2.00	8.60	58 ± 0.45	76 ± 1.94	80 ± 4.74
F18	30	1.00	8.50	42 ± 3.45	72 ± 3.37	76 ± 2.84
F19	65	2.00	7.00	55 ± 0.45	50 ± 1.97	54 ± 3.75
F20	100	1.00	5.50	41 ± 1.17	36 ± 2.26	40 ± 2.75

### 2.4 Evaluation of Prepared EN-TA-loaded Mesoporous Silica Nanoparticles

#### 2.4.1 Percentage Yield

The fully dried nanoparticles obtained were accurately weighed, and the percentage yield was calculated by the following equation:

$$\mathrm{Percentage}\;\mathrm{Yield}=\frac{\mathrm{Amount}\;\mathrm{of}\;\mathrm{Nanoparticles}}{\mathrm{Amount}\;\mathrm{of}\;\mathrm{drug}+\mathrm{Polymer}}\times 100.$$ (1)

#### 2.4.2 *In Vitro* Release of Econazole Nitrate and Triamcinolone Acetonide From Mesoporous Silica Nanoparticles

Release profiles of drugs from EN-TA-MSNs were accomplished using USP type-II dissolution apparatus (PT-DT7, Pharma Test, Germany). The drug-loaded MSNs were added in a dialysis sachet made of cellulose acetate membranes (Sartorius®, 0.45 μm pore size, MW-5000) containing 5 ml of dissolution medium. The dissolution medium consists of phosphate buffer having a pH of 6.4 at 37°C ± 0.5°C. The sachet was tied with peddle of the dissolution vessel by a heat resistant thread and set down in the dissolution medium (Katiyar et al., 2016). In order to determine drugs contents, about 5 ml of dissolution medium was taken out from every vessel at successive time intervals up to 14 days which was then supplanted by the same volume of freshly prepared medium in each and every vessel of the dissolution apparatus. An established and validated HPLC method was applied to determine the unknown concentration of EN and TA. The release of both drugs from MSNs was evaluated by applying five different kinetic models (Supplementary Table S1) in order to check the drug’s release mechanism.

### 2.5 Micromeritics Properties

Micromeritics deals with the flow properties of formulations and can be studied using various formulas, which are discussed below (Kiehlbauch et al., 2000; Gupta and Vyas, 2012). The accurately weighed amount of drug-loaded MSNs was first sieved and then transferred into a 100 ml graduated cylinder. The level of the EN-TA-MSNs was maintained carefully without the compacting and apparent volume was determined. Bulk density was calculated using the following formula:

$$Bulk\;density=\frac{weight\;of\;the\;MSNs}{volume\;of\;the\;MSNs}$$ (2)

An appropriate weight of drug-loaded MSNs was taken in a 100 ml graduated cylinder, which was then tapped 100 times to determine tapped density. The tapped density was calculated by using the following equation:

$$Tapped\;density=\frac{weight\;of\;the\;MSNs}{volume\;of\;the\;MSNs\;after\;100\;tappings}\;\;$$ (3)

Carr’s index (I) was calculated from the measurement of the tapped volume (vt) and the bulk volume (vb) using Equation (4).

$$I=\frac{Vb-Vt}{Vt\;x\;100}$$ (4)

The value of Carr’s index should be ˂20% for a good flow indication, and a value ˃20% specifies a poor flow character. The calculation of Hausner’s ratio was made from tapped density (ρt) and the bulk density (ρ*b*) of nanoparticles according to Equation (5).

$$Hausner\;ratio=\frac{\rho t}{\rho b}$$ (5)

It should be ˂ 1.25 for having a good flow character of nanoparticles. For the measurement of the angle of repose, an accurately weighed amount of nanoparticles was passed through the funnel on a plain paper sheet. Dropping nanoparticles created a pile on the paper sheet. The angle was calculated from the measurements of radius (r) and height (h) of the pile using Equation (6).

$$\tan\theta =\frac{h}{r}$$ (6)

The free-flowing behavior of the EN-TA-MSNs was confirmed by a value of less than 30°.

### 2.6 Entrapment Efficiency of EN-TA Loaded Mesoporous Silica Nanoparticles

For measurement of entrapment efficiency (EE), 30 mg of EN-TA-MSNs was weighed accurately, crushed, and then added to the 20 ml of phosphate-buffered saline (pH = 7.4). The resulting mixture was stirred (gently) for 24 h, followed by sonication for 15 min so that the entrapped drugs completely came out of MSNs. The mixture was filtered carefully, and after suitable dilutions, the drugs contents were determined by the HPLC method (Granero and Amidon, 2006). The EE was determined using the following equation:

$$Entrapment\;efficiency=\frac{Estimated\;drug\;content}{Theoratical\;added\;drug\;content}\times 100$$ (7)

### 2.7 Fourier Transform Infrared Spectroscopy

FTIR analysis was performed to monitor possible interactions between EN, TA, and EN-TA co-encapsulated MSNs. FTIR spectra were recorded with the Shimadzu ® IR prestige-21 instrument. The scan range was set between 400 and 4,000 cm^−1^ with a resolution of 4 cm^−1^ for 20 scans (Gupta and Vyas, 2012).

### 2.8 X-Ray Diffraction Analysis

X-ray diffraction analysis for pure drugs and MSNs loaded with EN-TA was performed using D8 advance X-ray (Bruker AXS, Madison, WI, United States) at 40 mA current using 40kV voltage. The sample was exposed by Cu-Ka generated monochromatic X-rays at the diffraction range (2*θ*) from 0 to 70^o^ at a speed of 20 s/min (Katiyar et al., 2016).

### 2.9 Thermal Analysis

Thermogravimetric analysis (TGA) and differential scanning calorimetry (DSC) of TA, EN, and MSNs loaded with EN-TA were performed by TGA/DSC-1 system (Mettler-Toledo, Switzerland). The samples were sealed in aluminum vessels, and thermal studies were performed from 40 to 300°C at a scanning rate of 20°C/min. During analysis, the inert atmosphere was ensured by running nitrogen at a rate of 50 ml/min (Katiyar et al., 2016).

### 2.10 Zeta Potential and Size Distribution Studies

Zeta potential and size distribution of EN-TA co-encapsulated MSNs were calculated by Zeta sizer Malvern version 7.11. For the size estimation, a 6 g sample was suspended in ultrapure deionized water containing 0.01% T-80 with the help of sonication process. A glass dish with MSNs suspension was then placed on a dynamic light scattering zeta sizer for obtaining measurements of size and charge. The analysis of the suspension was carried out at 25°C to determine the size, and the polydispersity index was calculated using the following formula:

$$Polydispersity\;index=\frac{Mw}{Mn}$$ (8)

where Mn is an average number and Mw is an average weight.

### 2.11 Scanning Electron Microscopy

The surface morphology of the optimized EN-TA–loaded MSNs was explored by scanning electron microscope (Mira-3 T Scan). For this purpose, the samples were prepared by sprinkling the nanoparticles on a double adhesive tape and then the adhesive tape was fixed on an aluminum fitting. The samples were gold coated under an inert atmosphere to make them electrically conductive (Zwiorek et al., 2004; Katiyar et al., 2016).

### 2.12 Biomedical Evaluation of Mesoporous Silica Nanoparticles

#### 2.12.1 Skin Irritation Testing

The study design for skin irritation, wound healing, and *in-vivo* antifungal activity was developed in compliance with the Helsinki declaration. Approval of the study design from the Ethical Committee of College of Pharmacy, University of Sargodha, Sargodha, Pakistan (EC N0: 106-May-20 for 6 months) was also obtained. One of the main disadvantages of the conventional use of EN-TA treatment is erythema or irritation of the skin, which sharply limits the application and tolerability of these drugs by patients. The comparative analysis of the skin-irritating potential of two drugs in suspension form and nanoparticles formulations was performed on rabbits (1.5–2.5 kg) through the Draize patch test (Draize, 1944). The rabbits were distributed into three groups, namely the control group (group I) that did not receive treatment, the group treated with EN-TA Suspension (group II), and the MSNs co-encapsulated EN-TA group (group III). The rabbits’ back hairs were shaved 24 h before application of suspensions and formulation. The formulations were uniformly applied on the hairless skin surface of 3 cm^2^. The applied amount of suspension (2.5 ml) and nanoparticles was equivalent to 150 mg of econazole and 5 mg of triamcinolone. The visible changes, such as erythema in the skin, were examined and measured on day 1, day 3, day 7, and day 14. An average value of three independent measurements was then taken as an erythema score for each rabbit under study. The mean erythema values which range from 0 to 4 were documented as follows: No signs of erythema = 0, minor erythema (light pink color) = 1, modest erythema (dark pink color) = 2, slightly severe erythema (light red color) = 3, and following severe erythema (intense redness) = 4.

#### 2.12.2 Cytotoxicity Studies

To evaluate the safety of formulated silica nanoparticles, the silica nanoparticles and their formulation ingredients were analyzed by cytotoxicity studies performed on breast cancer cell line (MCF-7), which were grown in Dulbecco’s modified eagle medium (DMEM) containing bovine fetal serum (10%). The Dulbecco’s modified eagle medium was consistently refreshed after every 48 h. The cells were supplied with DMEM without FBS just one day before the cytotoxicity study. In order to investigate the possible cytotoxic potential, dispersion (0.5%) of different samples was prepared in eagle medium and these dispersions were incubated for 6 hours and 24 hours in MCF-7 cells while keeping triton X-100 as the negative control and pure DMEM as the positive control. The MTT assay was performed on breast cancer cells after the completion of the incubation time period. The cells removed as samples from plates were washed thoroughly three times with isotonic phosphate buffer (PBS). Subsequently, 500 µL of a solution of MTT in DMEM without FBS (0.5 mg/ml) was added to each well, and the cells were incubated for another hour. Subsequently, the supernatants were eliminated. The converted dye was dissolved with 500 µL of DMSO and mixed well for a complete dissolution of the dye. This solution was then transferred into 1.5 ml tubes and centrifuged at 13,400 rpm for 2 min. The absorption of the resulting solution was recorded immediately at λ = 570 nm after dilution of the samples with an equal volume of DMSO. Subsequently, the supernatants were removed. The reacted dye was dissolved with 500 µL of dimethylsulfoxide and mixed well to completely dissolve the dye. The obtained solution was then taken in 1.5 ml tubes and centrifuged for 2 minutes at 13,500 rpm. The resulting solution was equally diluted with DMSO, and absorption of the resulting solution was recorded immediately at λ_max_ = 570 nm. By linking all values with 100% viability of the cells treated with DMEM, viability rates of the cells were calculated using the following equation:

$$Cell\;viability\left(\%\right)=\frac{As}{Ad}\times 100.$$ (9)

Here Ad indicates the value of absorbance taken after DMEM treatment, while As shows the value of absorbance taken after treatment with sample dispersions.

#### 2.12.3 *In Vitro* Antifungal Studies

To assess the antifungal properties of pure drugs and optimized formulation of EN-TA-MSNs, the process of diffusion test was utilized on the growth medium of Mueller Hinton agar by slight mediation in the procedure defined by the National Committee for Clinical Laboratory Standards and the *Candida albicans* were used in this study (Kiehlbauch et al., 2000). The *Candida albicans* cells were formed by the movement of a single colony of *Candida albicans* produced by the agar plates to sabouraud dextrose directly by inoculation under aerobic conditions at 35°C. The surface of the Muller Hinton agar plates has been inoculated with *Candida albicans* strains with the sterile cotton swab that was first soaked into the suspension of fungal species. The swab streaking was carried out over the entire surface of the agar plates, and then the process was repeated by rotating the agar plates by 60^o^. After this process, a cavity 3–4 mm thick was perforated, and a 50 µL EN-TA suspension and 50 µL MSNs loaded with EN-TA were added to different Petri dishes. The prepared agar plates were placed for 2–3 min at room temperature and then incubated for 24 h at 35°C. The zone of inhibition as an indicator of decline in growth density of *Candida albicans* was measured using Vernier calipers.

#### 2.12.3 *In Vivo* Antifungal Studies

Rabbits (1.5–2 kg) were divided into three different groups, each consisting of six rabbits. *Candida albicans* (MTCC 183) was used to induce fungal infections. *Candidiasis* was induced on the skin of the rabbits by a slight change in an already described procedure (Gupta and Vyas, 2012). The hairs on an area of 4 × 4 cm^2^ on the rabbit’s back were removed using an anti-hair cream. This skin area was then marginally scratched with sandpaper on the next day, and previously prepared *CA*-inoculum (600 mg) was applied using a glass rod. Suspension of pure drugs and EN-TA–loaded silica nanoparticles were applied to the rabbits in two groups for 14 days, starting from the day following infection, with the exception of the rabbits in the control group. The rabbits of the control group were also infected but did not receive any treatment. After the start of treatment, all the rabbits were visually monitored for any change in the skin structure of the infected area without any interruption. The antimycotic activity of the other two groups was compared with the control group. and the treatment time interval along with the changes in the skin texture was studied. Culture studies were conducted to evaluate the effectiveness of treatment. The area of the skin was removed from the treated area, crumbled with scissors, and homogenized with a tissue homogenizer in 4 ml of saline solution. Part of the homogenate was streaked on the solidified agar chromium media, and Petri dishes were then incubated at about 25°C in the incubator. The counting of the number of colony-forming units (CFUs) was performed on the agar plates, and the logarithm of the number of CFUs was designed for all infected sites. The rabbits were considered as a positive fungus after observing more than one fungal colony.

#### 2.12.4 Wound Healing Studies

Before the experiments, a group of male and female rabbits aged 8 weeks (1.5–2 Kg) were individually placed in hygienic cages for 2 weeks under controlled conditions (at 20–25°C, 12 h dark-light cycle, and 65–70% relative humidity) in order to have time for acclimatization. The rabbits’ backs were trimmed hairless 24 h before the treatment. The hairless area was cleaned without germs with a mixture of ethanol-iodophor (76%, 4%), and a complete thick excision was surgically made on the hairless skin. After marking a circular surface area of 2 cm with methylene blue, the rabbits were anesthetized for this operation by using an IV injection of sodium pentobarbital, and the removal of the skin in full thickness was carried out using incision scissors. The rabbits were then randomly assigned to either a control group (*n* = 24) or the EN-TA-MSNs treatment group (*n* = 24). The injured area was topically treated with Vaseline and coated with a surgical gauze reinforced with a bandage in the control group. On the other hand, in the treated group, after excision, the topical wound was immediately protected with the EN-TA–loaded MSNs and fixed with a paragon bandage. The injured areas of both groups were comparatively evaluated, and their images were captured at different time intervals (first, third, 7th, and 14th days).

#### 2.12.5 Histopathological Studies

Tissue samples of the injured areas (control and treated groups) of about 1.5 cm sampled on four different days (1st, 3rd, 7th, and 14th days) were preserved for about 24 h in a solution of formaldehyde (3.7%) at pH 7.4. Subsequently, dehydrated and purified samples with a thickness of about 3 μm were placed and stained on separate glass slides. The segments of tissue from wounded areas were then analyzed, and the images were obtained with a light microscope.

## 3 Results and Discussions

### 3.1 Statistical Optimization of Percentage Yield and Drugs Release From Mesoporous Silica Nanoparticles

It was found that the percentage yield of silica nanoparticles was in the range of 33–88%, with formulation F11 having the maximum yield of 88%, while formulation F3 had the minimum yield of 38%. The increase in oil concentration increased the percentage yield of MSNs, as shown by the formulations F11 and F14. Similarly, the increase in the stirring time also led to increased percentage yield, as exhibited by the formulation F11, which indicated that a sufficient stirring time is required to produce nanoparticles in an appropriate amount of vegetable oil that actually serves as a medium for the production of nanoparticles, while the effect of pH on the percentage yield is minimal among three variables studied. It is proved that the two independent variables such as the vegetable oil concentration and the stirring time have a dominant influence on the percentage yield (Nandy and Mazumder, 2014). The data for percentage yield has followed the linear model as shown in Supplementary Figure S1, and the software generated polynomial equation is as follows:

$$Percentage\;Yield=-23.179+1.839X_{1}\;+9.532X_{2}+6.776X_{3}$$ (10)

It is obvious that all formulation variables have a positive effect on the percentage yield of MSNs, and in order to improve the MSNs yield, a higher level of studied formulation variables would certainly produce a positive effect. But the impact of stirring time was found to be more prominent as compared to vegetable oil concentration and pH level because the coefficient value of X_2_ (9.5) is comparatively higher. A higher stirring time led to an increase in PY to more than 70% (F13), which could be due to the fact that longer time of stirring leads to greater diffusion of solvents, which leads to an increase in PY and, conversely, lower stirring time and PY. Likewise, increasing the stirring time also leads to smaller nanoparticle size. The outcomes of ANOVA (Table 2) for percentage yield have also suggested these variables as significant (Huang, 2007; Matlhola et al., 2015).

**TABLE 2:** Results of ANOVA for responses Y_1_, Y_2_ and Y_3_ from EN-TA loaded MSNs.

Factors	Parameters					
	Percentage yield		Release of EN		Release of TA	
	*p*-Value	f-Value	*p*-Value	f-Value	*p*-Value	f-Value
Model	22.11	33.70	<0.0001	586.94	<0.0001	488.69
X_1_-Oil concentration	<0.0001	3.95	<0.0001	0.0196	<0.0001	4.33
X_2_-Stirring time	<0.0001	0.7387	<0.0001	0.0005	<0.0001	21.37
X_3_-pH	<0.0001	—	<0.0001	<0.0001	<0.0001	4,127.5
X_1_X_2_	—	—	0.1351	0.1351	0.0272	6.68
X_1_X_3_	—	—	0.0349	0.0349	0.0023	16.49
X_2_X_3_	—	—	1.0000	1.0000	0.0947	3.41
X_1_^2^	—	—	0.1461	0.1461	0.0672	4.21
X_2_^2^	—	—	0.1773	0.1773	0.4727	0.5569
X_3_^2^	—	—	<0.0001	<0.0001	<0.0001	196.51

Initially, a slow drug release was observed in *in vitro* release studies of MSNs. The amount of drug release from MSNs slowly increased over time, which showed the prolonged release of drugs from nanoparticles, as shown in Supplementary Figure S2 (Kumar et al., 2014). *In vitro* release studies displayed a better control of release over a longer period of time (Jain et al., 2010). The release of EN and TA from the MSNs ranged from 30 to 78 and 35%–84%, respectively (Table 1). The formulation F11 exhibited a maximum release of EN (68%) and TA (74%), respectively, while the formulation F1 demonstrated a minimum release of EN (30%) and TA (35%), respectively. MSNs formulated at optimal oil concentration with a reasonable stirring time presented better control of the release of both drugs. Increasing the stirring time to a very high level led to cracks in the MSNs, which eventually increased the release of drugs. Similarly, MSNs formulations with optimal pH also have decrease in drug release, which could be associated with a small porous volume of silica nanoparticles formulations (Huang, 2007). Similarly, a high pH leads to an increase in the pore volume, which leads to a faster release of the drug from the formulation. The presence of enlarged and large pores leads to greater penetration of water into the nanoparticles, which would further improve the release of both drugs (Cooper and Harirforoosh, 2014). The polynomial equations generated by software design expert for the release of EN and TA are as follows:

$$EN\;\mathrm{Release}=70.368-0.267X_{1}-2.849X_{2}-16.343X_{3}+0.148X_{1}X_{2}+0.148X_{1}X_{3}+3.299X_{2}X_{3}+0.782X_{1}^{2}+0.897X_{2}^{2}+1.997X_{3}^{2}$$ (11)

$$TA\;\mathrm{Release}=94.966-0.359X_{1}-5.019X_{2}-20.161X_{3}+0.251X_{1}X_{2}+0.269X_{1}X_{3}+0.467X_{2}X_{3}+0.121X_{1}^{2}+0.469X_{2}^{2}+2.577X_{3}^{2}$$ (12)

On drug release, the effect of all three studied variables at their optimum levels remained negative suggesting that their optimum level would yield MSNs which could make the availability of drugs slow and sustained for a prolonged time. However, to study the *in-vitro* drug release, pH seems to be a crucial factor that decides the dissolution fate of the formulation. The polynomial values clearly showed that X_3_ (16.30443) had a significant antagonistic effect on the release of EN since the value of its coefficient was higher than that of the other variables X_1_ (0.206, 571) and X_2_ (2.84596). Similarly, for the release of TA, polynomial values showed that X_3_ (20.16142) had a significant antagonistic effect since the value of its coefficient was higher than that of the other variables X_1_ (0.359,552) and X_2_ (5.01925). The interactions terms X_1_X_2_ (0.014286), X_1_X_3_ (0.014286), and X_2_X_3_ (3.29494) were found to be synergistic because of their positive signs. For TA release, the interactions X_1_X_2_ (0.025000), X_1_X_3_ (0.026190), and X_2_X_3_ (0.416,667) were synergistic interactions. It was also observed that a very high level of vegetable oil (X_1_^2^), stirring time (X_2_^2^), and pH (X_3_^2^) seemed to be unsuitable for controlling drug release because they showed positive influence/signs suggesting that they would lead to the enhancement of release of drugs from MSNs. Factor X_3_^2^ (1.99427) showed a significant effect as its value was greater than X_1_^2^ (0.000782) and x_2_^2^ (1.99427). The data on the release of the two drugs best corresponds to the quadratic model presented in the 3D graphs in Supplementary Figure S1. The statistical analysis (Table 2) demonstrated that the three variables studied turned out to be significant (<0.0001), not only for the yield of MSNs but also for the release of EN and TA from the formulations of MSNs. The study indicated that the release of drugs does not merely depend on the single formulation parameter since the three studied parameters such as oil concentration, pH, and stirring simultaneously affect the results of the release of the two drugs. The drug release from suspension was found to be rapid and fast, and the whole amount of both drugs was available within 3 hours. The drug release profiles were analyzed in order to find out the release mechanism by applying different kinetic models (first order, zero order, Korsmeyer–Peppas, and Higuchi models). In this analysis, the values of rate constants (K), release exponent (*n*), and correlation coefficients (*R* ^*2*^) obtained from studied models were comparatively analyzed. The release kinetic profiles were best suited to zero order because the value of the correlation coefficient (*R* ^*2*^) was found to be higher and close to unity (Supplementary Table S2) which indicated that drugs release was independent of the remaining amount in silica nanoparticles. In the Korsmeyer–Peppas model, the value of *n* indicated that the release mechanism followed a slow diffusion along with mild erosion of nanoparticles.

The values of the correlation coefficients (*R* ^*2*^) were found at 0.8056 for PY, 0.9981 for the release of EN, and 0.9977 for the release of TA. The obtained outcomes were observed to be very close to the adjusted *R* ^*2*^ results, indicating the importance of the model used. The ratio between the maximum and the minimum for Y_1_, Y_2,_ and Y_3_ was observed at 3.2, 3.72, and 3.43, respectively, which indicates that it was not necessary to change the model further since it turned out to be 3. The applied model was considered significant because the *p*-value was <0.05. The quadratic model was followed by the three answers because their data depicted a good connection with the quadratic. The signal-to-noise ratio was measured with adequate precision, and its results of 19.73 for Y_1_, 77.25 for Y_2,_ and 71.64 for Y_3_ were observed as > 4 (Table 3), which indicates the adequacy and suitability of the quadratic model applied for dependent variables. The actual experimental results for the percentage yield and drug release were very close to the expected results proposed by the CCRD (Supplementary Figure S2).

**TABLE 3:** Fit statistics data analysis for the effect of selected variables on the responses.

Variables	Y_1_	Y_2_	Y_3_
Std deviation	6.45	0.8699	0.9577
Mean	57.00	52.80	56.95
C.V%	11.31	1.65	1.68
*R* ^*2*^	0.8056	0.9981	0.9977
Adjusted *R* ^*2*^	0.7692	0.9964	0.9957
Predicted *R* ^*2*^	0.5762	0.9743	0.9759
Adeq precision	19.7391	77.2559	71.6463

In order to further validate and optimize the MSNs, a batch of optimized nanoparticles was manufactured using the recommended optimized levels of formulation factors (Table 4) as suggested by software Design Expert (version 8.0.6.1 Stat-ease, Inc., United States). The optimized formulation of MSNs has been established to achieve a higher PY (85%) and a controlled release of active ingredients (50%) using the process of numerical optimization. The proposed variations of formulation conditions and desired results were prioritized according to the desirability factor, and for all MSNs-dependent variables, the desirability factor was observed close to unity. Thus, software generated and optimized drug-loaded MSNs have been formulated with a zeta potential of -25 mV and mean size of 450 nm (Table 4) and have been analyzed for different biomedical applications. The formulated optimized MSNs showed a PY of 85% and the release of active substances of EN and TA (68 and 70%).

**TABLE 4:** Level of components, experimental versus predicted results of PY, EN release, TA release, desirability factor, size, and ZP of optimized MSNs.

Composition of optimized MSNs		MSNs responses	Exp. value	Predicted value	DF	Size (nm)	ZP (mv)
Oil concentration (RPM)	100	PY	85.3	88	0.891	450 ± 5.72	-25 ± 1.23
Stirring time (min)	120	EN release	68.5	75.5	0.937	—	—
pH	5.6	TA release	70.5	75.5	0.906	—	—

Exp: Experimental, PE: prediction error, DF: desirability factor, ZP: zeta potential.

### 3.2 Micromeritics

Micromeritics deals with the analysis of the flow properties of a system. The appropriate flow rate of the drug-loaded MSNs is necessary if they are to be transformed into tablets, capsules, transdermal patches, or any other dosage form for comfortable administration to patients. The studied parameters strongly influenced the flow properties of MSNs formulations. The results of Carr’s index for all MSNs formulations ranged from 9 to 20, which indicated a better flow of formulated MSNs (Supplementary Table S3). The angle of repose had also confirmed the good fluidity of EN-TA-MSNs since the value of the angle remained < 22°. Similarly, the results of Hausner’s ratio for all formulations remained below 1.5, which indicates the excellent micromeritics of drug-loaded MSNs (Kiehlbauch et al., 2000).

### 3.3 Entrapment Efficiency of EN-TA Loaded Mesoporous Silica Nanoparticles

The entrapment efficiency (EE) of both drugs in MSNs was observed to be within a range of 35–82% (Supplementary Table S3). There was a prominent increase in the EE of both drugs with an increase in stirring time, pH, and vegetable oil concentration as observed for F1, but a very level of these factors (F11 and F13) had a prominent fall in EE was observed. The decreased EE at the higher stirring time was due to the displacement of particles toward the walls of the flask, and consequently, the contact time between polymer and drugs was reduced. Similarly, a prominent decline in EE of both drugs was observed at the decreased level of stirring time and lower concentration of vegetable oil (F18). The formulations (F5) formulated at an increased pH value have a higher EE of more than 50% that could be associated with higher stirring time and an increased level of oil. The highest EE (79% for TA and 82% for EN) was observed in formulation F1 prepared at a stirring time of 2 h as compared to formulation-F18 prepared at a lower stirring time of 1 hour. It may be associated with the availability of sufficient time for proper tightening of the silica network that may not be possible at a lower stirring time. The EE for both drugs for formulated MSNs at the lower stirring time was found to be low because aggregation and coalescence become more and more evident at reduced stirring speed.

### 3.4 Zeta Size and Zeta Potential Analysis

**FIGURE 1:**
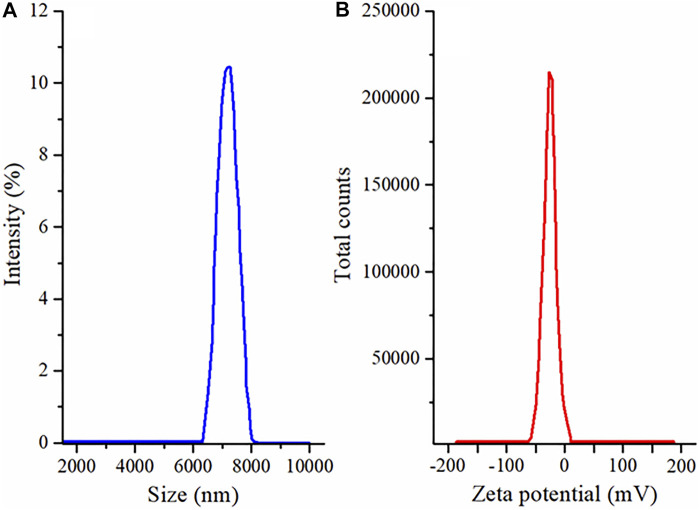
**(A)** Size distribution and **(B)** zeta potential analysis of EN-TA loaded MSNs.

To determine the physical stability of the MSNs, particle size is a good indicator. The zeta size of optimized EN-TA–loaded MSNs exhibited a single peak with an intensity of 100% at 416.8 nm, as presented in Figure 1A. It was observed that the average particle size of the EN-TA charged MSNs was less than 480 nm. Particle size plays an important role in the administration of the drug since small particles can easily and quickly cross the skin barrier layers compared to larger particles. In addition, a reduced particle size certainly leads to a larger area which increases the solubility of drugs. Therefore, the size range of the optimized nanoparticles proved to be sufficient and remained in accordance with the specifications (Table 4). The value of the poly dispersibility index was observed at 0.49, which indicates that the system of administration of the drugs has a fairly uniform size distribution. The nanoscale size of MSNs also proves the stability of EN-TA–loaded MSNs, which could explain the relevance of the administration of MSNs since the stable MSNs are easier to delay and prolong the administration of the drugs at the site of application (Kiehlbauch et al., 2000).

As shown in Figure 1B, the zeta potential of the MSNs loaded with EN-TA had a single net peak at −25 mV covering 100% of the area, which may be due to the presence of negatively charged silica (-OH) silanol groups. It was observed that the co-encapsulated EN-TA MSNs were stable and that no signs of aggregation appeared since the zeta potential was within the defined limit. A formulation is more stable when all particles are positively or negatively charged, instead of some particles having negative potential and some particles having positive potential, because the opposite charges attract each other and are considered less stable. In addition, there are repulsive forces between the same charges, which keeps the formulation stable for a long time (Tummala et al., 2015; Bahl, 2017).

### 3.5 X-Ray Diffraction Studies

**FIGURE 2:**
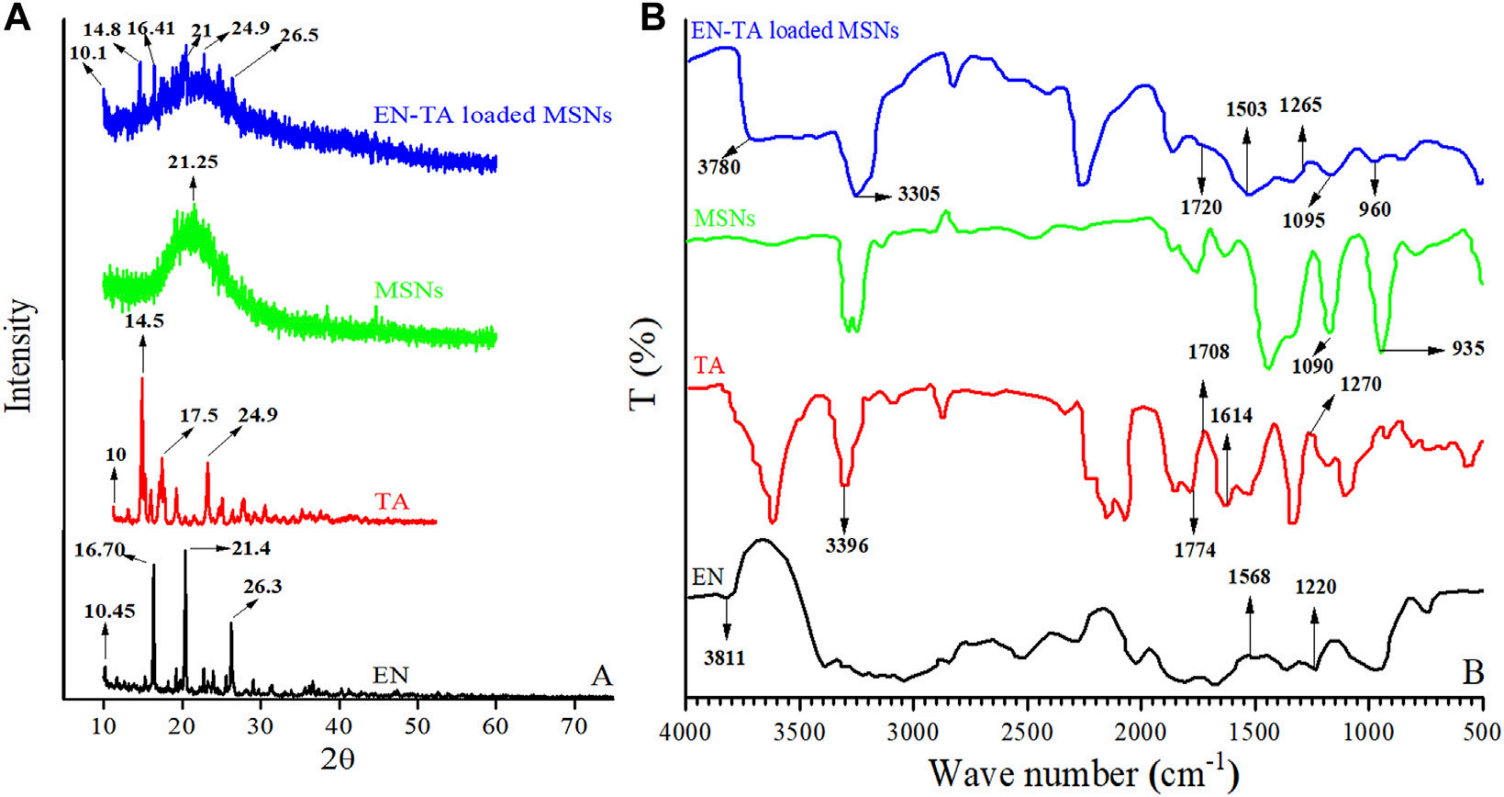
**(A)** XRD and **(B)** FTIR spectra of econazole nitrate (EN), triamcinolone acetonide (TA), MSNs, and EN-TA loaded MSNs.

XRD is an expedient technique involved to investigate the degree of crystallinity of drugs after their inclusion into the formulation. The XRD peaks depend mainly on the particle crystal size, as they indicate the crystal value at a given value in the 2-theta range. In the current study, the XRD pattern of EN (pure drug) exhibited three main peaks at 10.45, 16.70°, 21.4°, and 26.3°, indicating its crystalline nature, and smaller peaks were also observed (Abd El-Gawad et al., 2017), as shown in Figure 2A. Similarly, the XRD of TA showed four main peaks at 10, 14.5°, 17.5°, and 24.7°, which revealed the crystalline structure of the pure drug (Ho et al., 2019), and some light peaks were also observed in the XRD results of TA, as shown in Figure 2A. Significant changes in the intensity of the EN and TA peaks have obviously been observed in the diffractogram of EN-TA–loaded optimized MSNs. Changes in the diffractograms may be attributed to the distribution of pure drugs in the pores of MSNs, which reduces the level of detection. In addition, the slight depression of the intensity of the pure drugs peaks highlighting the fact of inclusion of drugs in silica nanoparticles (Tummala et al., 2015).

### 3.6 Fourier Transform Infrared Analysis

FTIR analysis of pure drugs (EN and TA), MSNs, and optimized formulation (EN-TA-MSNs) were performed to explore the possible interactions between the drugs and carriers. In the case of pure EN, the FTIR spectrum showed absorption at wave number 3811 cm^−1^, which indicated the stretching of the -NH groups. Stretching frequencies observed at 1568 cm^−1^ suggested the presence of the NO_2_ group in EN. The functional groups C-N were indicated by the stretching peaks that appeared at the absorption wave number 1220 cm^−1^ (Figure 2B). Likewise, spectra of pure TA exhibited a typical absorption band at wave number 3396 cm^−1^ associated with the hydrogen-bonded hydroxyl group (-OH). A peak that appeared at 1708 cm^−1^ was related to the C=O (keto) group which is also visible in spectra of drug-loaded MSNs. The existence of aliphatic ester group was identified by absorption frequency observed at 1774 cm^−1^. The stretching frequencies at 1614 cm^−1^ and 1270 cm^−1^ indicated C-H functional groups and C-O-C bond of the aliphatic esters were observed in the spectra of pure TA and in the case of drug-loaded MSNs (Figure 2B). FTIR of MSNs exhibited a characteristic peak of Si-O-Si at 1050 cm^−1^ and a shoulder at 940 cm^−1^ representing free–OH (Shafqat et al., 2019).

It was observed that the FTIR spectrum of the EN-TA–loaded MSNs did not exhibit any new peak. Furthermore, it was found that there were no significant shifts in the absorption peaks of the drugs, which indicated that there is no structural interaction between drugs and formulated MSNs (Abd El-Gawad et al., 2017; Ho et al., 2019).

### 3.7 Thermal Analysis (Thermogravimetric Analysis and Differential Scanning Calorimetry)

**FIGURE 3:**
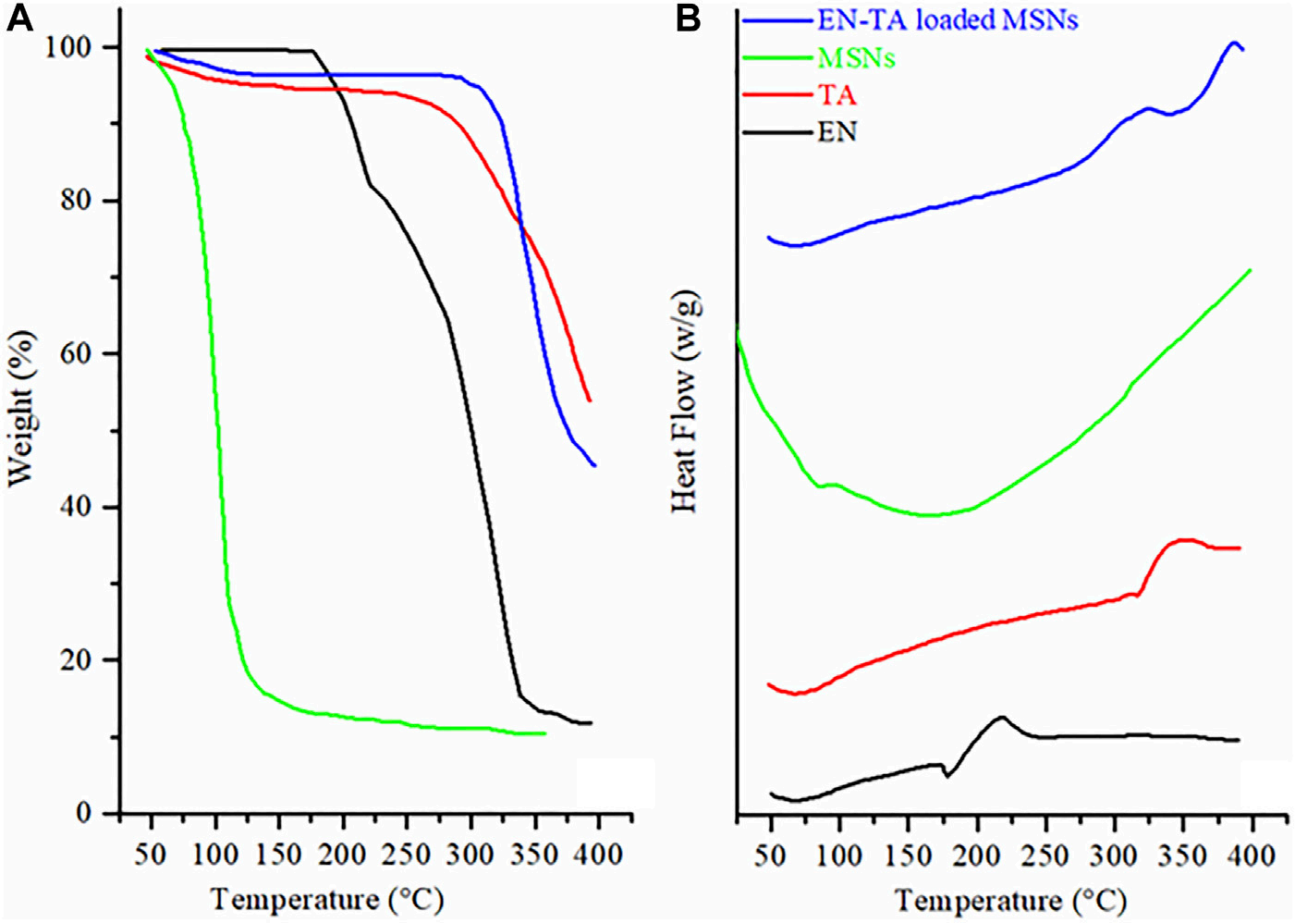
**(A)** TGA and **(B)** DSC thermograms of econazole nitrate (EN), triamcinolone acetonide (TA), MSNs, and EN-TA loaded MSNs.

TGA was conducted to calculate the encapsulated amount of EN and TA in MSNs and DCS was performed to ascertain the presence or absence of the crystalline form of drugs in the pores of the carrier. TGA curve of EN revealed weight loss at two-point. The first weight loss of 17.05% was observed at 218.71°C and the second major weight loss of 66.22% was observed at 335.82°C, as presented in Figure 3A. The initial weight loss may be linked to loss of moisture, and the huge weight loss may be associated with the degradation of drug. While the DSC of EN showed two pronounced peaks at 177.53°C and 250°C. The pronounced peak located near the melting point of EN represented the crystalline nature drug. The thermogram of TA exhibited weight loss in two stages. Initially, a weight loss (6.47%) found at 306.60°C was linked to the loss of moisture. The second weight loss of 42.93% was due to the decomposition of drugs. While the DSCs of TA exposed a peak at 316.40°C near the melting point of TA is an evidence of the crystalline nature of drug (Abd El-Gawad et al., 2017; Ho et al., 2019).

Thermal analysis of the EN-TA–loaded MSNs revealed weight loss in three different points. The first weight loss of 4.66% observed at 94.27°C was linked to the removal of moisture. Another minor weight loss (4.04%) at 288.12°C, may be linked to the decomposition of organic functional groups. The third major weight loss of 23.36% observed at 381.01°C was due to the degradation of drugs. The highest weight loss observed in case of EN-TA–loaded MSNs evident maximum encapsulation of both of the drugs in the MSNs pores. The drug-entrapped values measured by HPLC and shown in Supplementary Table S3 were in line with those obtained by thermal analysis. The EE of optimized formulation of EN-TA–loaded MSNs for both drugs was 79% for TA and 82% for EN. The findings indicated that the drug entrapment efficiency of MSNs is primarily due to porosity of MSNs.

While DSC of the EN-TA–loaded MSNs revealed three peaks. The two exothermic peaks were due to the degradation and pyrolysis of drugs and MSNs (Figure 3B). The single endothermic peak was observed at 74°C, which was due to residual water loss. Melting point peaks of EN and TA were absent in the DSC curves of drug-loaded MSNs. The absence of melting point peaks is a clue toward the phase transition of drugs, and drugs have been merged into the pores of silica in amorphous form (Feng et al., 2014). DSC outcomes are in agreement with the finding of XRD (Jain et al., 2010; Helal et al., 2012).

### 3.8 Scanning Electron Microscopy

**FIGURE 4:**
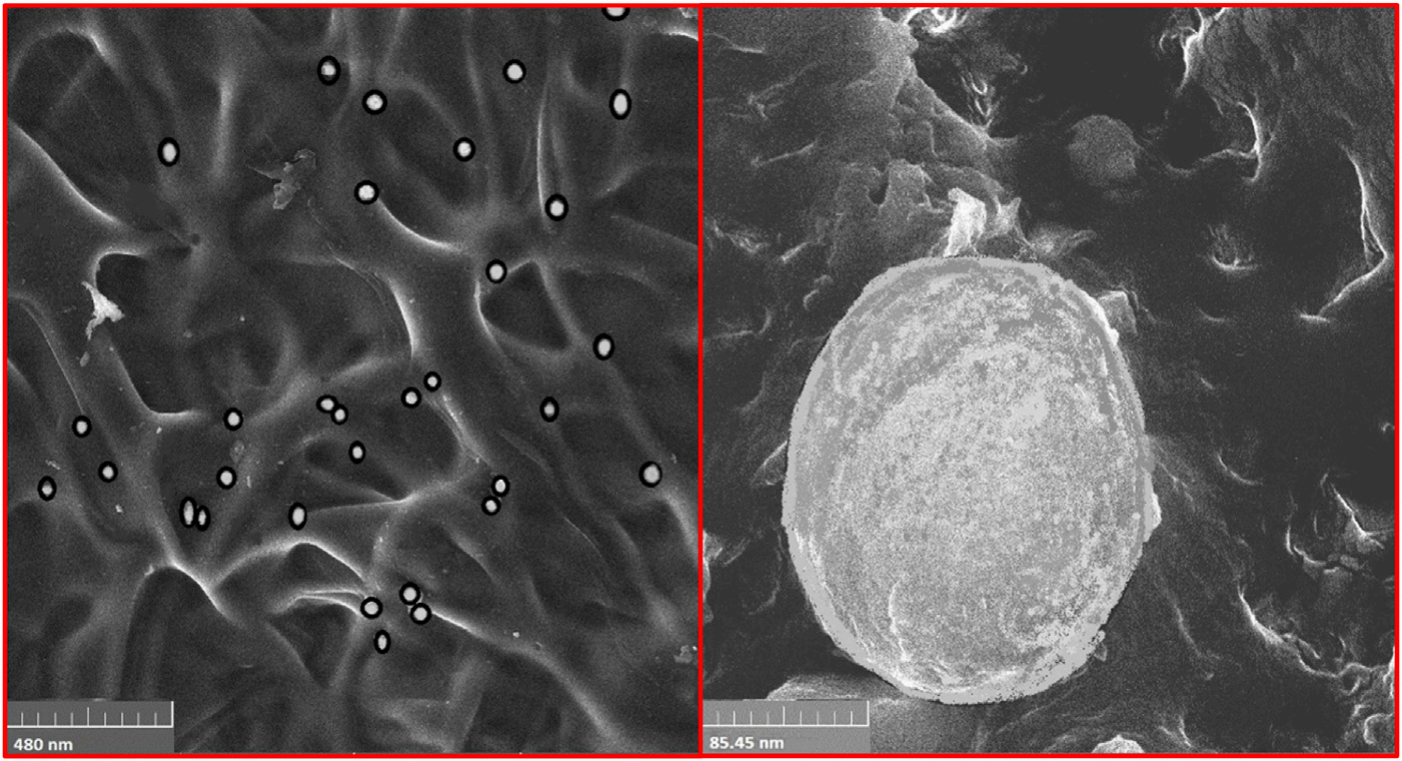
SEM analysis of EN-TA-MSNs.

The SEM analysis was performed to determine the surface morphology of the co-encapsulated EN-TA MSNs. The SEM analysis of MSNs confirmed the porous structure of MSNs, which is the most favorable for cell proliferation, cell attachment and cell migration (García-Millán et al., 2017). In addition, the porous structure also promotes the supply of oxygen. It was observed that the MSNs loaded with EN-TA are exclusively uniform, monodispersed, and spherical in shape having an average particle size of about 500 nm (Figure 4) (Gao et al., 2009).

### 3.9 Biomedical Evaluation of Mesoporous Silica Nanoparticles

#### 3.9.1 *In Vitro* Antifungal Studies

**FIGURE 5:**
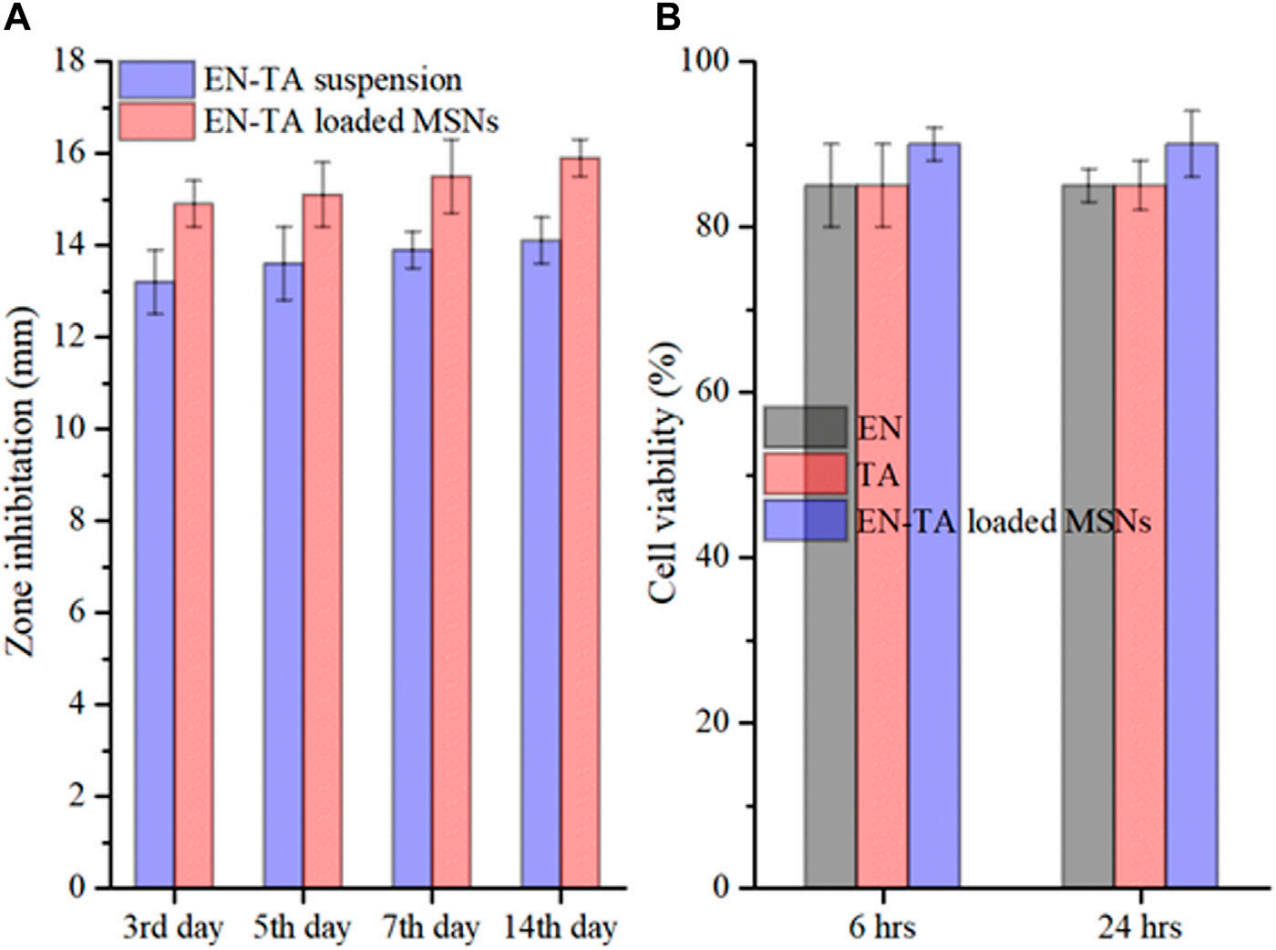
*In vitro* antifungal study **(A)** and cytotoxicity study **(B)** of pure EN-TA suspension and EN-TA loaded MSNs.

To determine *in vitro* antifungal activity, pure EN-TA suspension and EN-TA–loaded MSNs were tested, and inhibition zones were calculated on the third, fifth, 7th, and 14th days (Lee et al., 2011). A substantial difference was recorded between the areas of inhibition of the two formulations. On the third day, EN-TA suspension represented a 13.20 mm inhibition zone and EN-TA–loaded MSNs exhibited 14.80 mm. On the fifth day, the inhibition zone was 13.60 and 15.10 mm for EN-TA suspension and EN-TA–loaded MSNs, respectively. On the seventh day, the inhibition zone was exposed to 13.90 mm by EN-TA suspension and 15.50 mm by EN-TA–loaded MSNs. On the 14th day, the inhibition zone was 14.10 mm for the EN-TA suspension and 15.90 mm for the EN-TA–loaded MSNs, as shown in Figure 5A. According to the results, it was easily predicted that the antifungal activity of co-encapsulated econazole-triamcinolone MSNs was absolutely superior to the pure econazole-triamcinolone activity (EN-TA suspension) due to a larger inhibitory zone and that the drug-loaded MSNs would be better accepted as compared to the pure suspension (Ambrogi et al., 2010; Ninan et al., 2013).

#### 3.9.2 Cytotoxicity Studies

The cytotoxicity of EN, TA and co-encapsulated EN-TA-MSNs was studied in 96-well plates using a breast cancer cell line (MCF-7 cells). After 6 h, the cells treated with EN and TA showed about 85% survival and about 90% survival for the co-encapsulated EN-TA-MSNs (Figure 5B). The cell viability percentage of EN and TA was 85%, and the cell viability percentage was 90% for MSNs even after 24 h. The results indicated that the survival percentage of the cells after 24 h was not significantly different from the survival percentage of the cells after 6 h. These cytotoxicity studies showed that the percentage of cell survival was higher for EN-TA-MSNs as compared to pure drugs, which showed that drug-loaded MSNs were less cytotoxic in contrast to pure drugs (Zwiorek et al., 2004).

#### 3.9.3 Skin Irritation Studies

**FIGURE 6:**
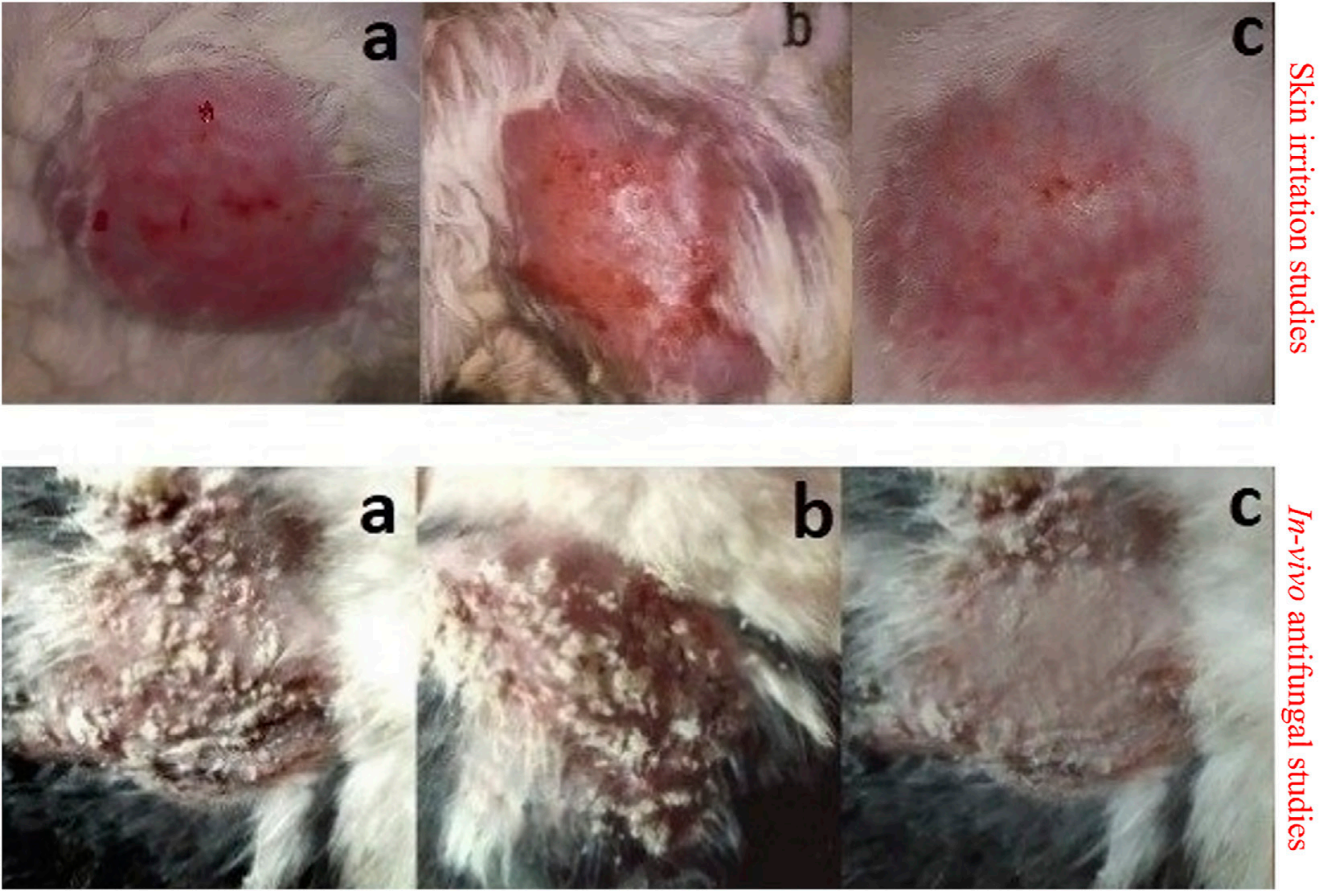
Skin irritation studies and *in vivo* antifungal studies in control group **(A)**, group treated with pure EN-TA suspension **(B)**, and group treated with EN-TA loaded MSNs **(C)**.

One of the main shortcomings of EN/TA treatment is erythema or skin irritation, which severely restricts its application and tolerability for patients. Ideally, the EN-TA drug delivery system should be capable of reducing or eliminating these erythematic incidents. It was assumed that after the encapsulation of EN-TA drugs in MSNS, the continuous interaction of EN-TA with the stratum corneum (the activating feature of erythematic episodes) of the skin would decrease, which would lead to fewer erythematic events. The results of the current skin irritation study are listed in Table 5. The study specified that when EN-TA were encapsulated in MSNs, the resulting formulation caused significantly less irritation as compared to EN-TA suspension even after 14 days of application. In addition, a continuous increase in erythema was observed in the EN-TA suspension treated group (Figure 6). This study demonstrated the extraordinary advantage of EN-TA-MSNs over drugs in suspension form, thereby confirming the improvement of tolerance to the skin for topical administration of EN-TA, which would be helpful in refining patients’ compliance (Jain et al., 2010).

**TABLE 5:** Mean erythema scores found for various formulations and *in vivo* antifungal studies in three groups of rabbits.

Sr. No.	Formulation and treated groups	Mean erythema scores			*In vivo* antifungal efficacy	
		1st Day	7th Day	14th Day	Rabbits having positive test/Total no of rabbits	Infected sites/Log CFU
1	Group II (Control group)	0	0	0	6/6	4.47 ± 0.61
2	Group II (EN-TA suspension)	1	3	4	4/6	3.53 ± 0.28
3	Group II (EN-TA loaded MSNs)	0	1	0	1/6	0.21 ± 0.15

#### 3.9.4 *In Vivo* Antifungal Studies

*In vivo* antifungal effect of pure drugs suspensions and co-encapsulated EN-TA-MSNs was evaluated in a rabbit model. MSNs loaded with EN-TA proved to be effective in eradicating *candidiasis* because only a single animal presented a positive culture test, while four animals out of six exhibited a positive culture test in the control group (EN-TA suspension treated group) as presented in Table 5. A positive culture test was observed for all rabbits in the control group without treatment (Figure 6). A fast recovery from infections was observed in drug-loaded MSNs and such high effectiveness of MSNs was most likely due to improved bioadhesive, occlusive, and controlled release of the drug (Jain et al., 2010).

#### 3.9.5 Wound Healing Studies

**FIGURE 7:**
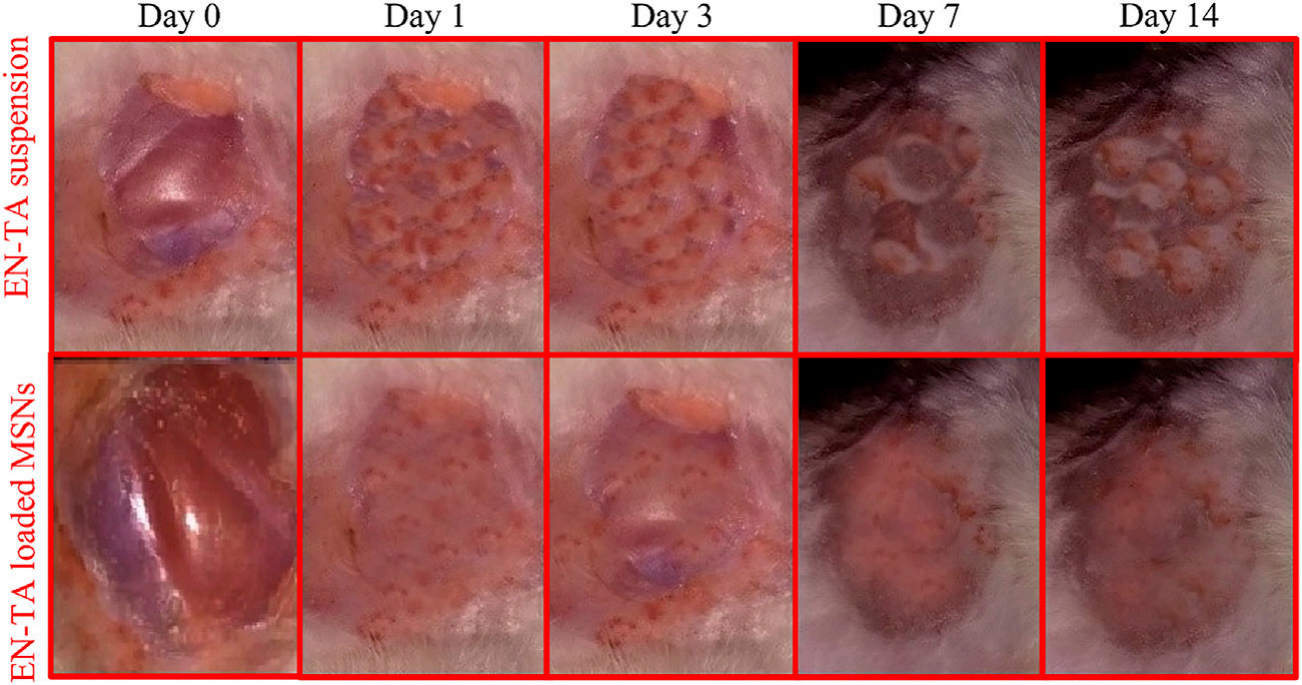
The wound healing efficacy of pure EN-TA suspension (control group) and EN-TA loaded MSNs treated group.

The optimized formulation of EN-TA-MSNs and pure drugs suspensions were evaluated for their wound healing properties by observing morphological changes exhibited in the full thickness skin infected model, as presented in Figure 7. After 24 h, it was observed that in the case of drug-loaded MSNs treated rabbits, the injured skin exhibited rapid repair of the damaged tissues. In the control group, the skin area near the wound showed inflammation, but the injured area of the skin was slightly reduced. On the third day, the surface of the injured skin in the EN-TA-MSNs treated group was observed to be dry, the inflammation around the injured skin gradually subsided, producing a layer of pale yellow crust, and the injured area was sufficiently decreased. The wound in the EN-TA suspension treated group (control group) remained circular, inflamed, and without crust to a greater extent. In addition, in the treated group, the wounds were observed to have an irregular oval or circular shape with a soft consistency, and wound diameter was also lesser than in the control group. On the seventh day of the injury, the inflammation in the treated group was completely disappeared, and the injured edges were obsolete. On the other hand, in the control group, wound color was observed to be pale with signs of inflammation, and the injured area was more stable and deeper. On the 14th day, it was observed that, in the treated group, the injured area was almost absolutely healed. There is no doubt that the EN-TA-MSNs treated rabbits exhibited an accelerated wound healing efficacy with little or no crust on day-14 (Ambrogi et al., 2010).

#### 3.9.6 Histopathological Studies

Wound healing is a slow process. In this regard, external wound dressing is ever required to regulate and accelerate the process. To observe the wound healing efficacy of pure drugs and drug-loaded MSNs, the wounded tissues were stained with EN-TA suspensions and EN-TA-MSNs. On the first day, signs of accumulation of sheets of the extracellular matrix along with bleeding in the group treated with EN-TA suspension were clearly observed, and the accumulation of macrophages was also considerably lower compared to the MSNs treated group. In the MSNs treated group, the space filled by the blood clot and a small number of sheets resembling the appearance of the extracellular matrix (ECM) was also obvious and prominent. A thick sheet of ECM was observed in the control group on the third day. Very few macrophages were observed, so the ECM clearance rate was slower in the control group (treated with EN-TA suspension), while in the group treated with MSNs, an extracellular matrix accumulation was observed in the damaged tissue, a red color extracellular matrix was infiltrated by macrophages, which in turn triggered the extracellular matrix clearance, which resulted in parallel fibers. It became clear that the healing process was most evident in the MSNs treated group.

**FIGURE 8:**
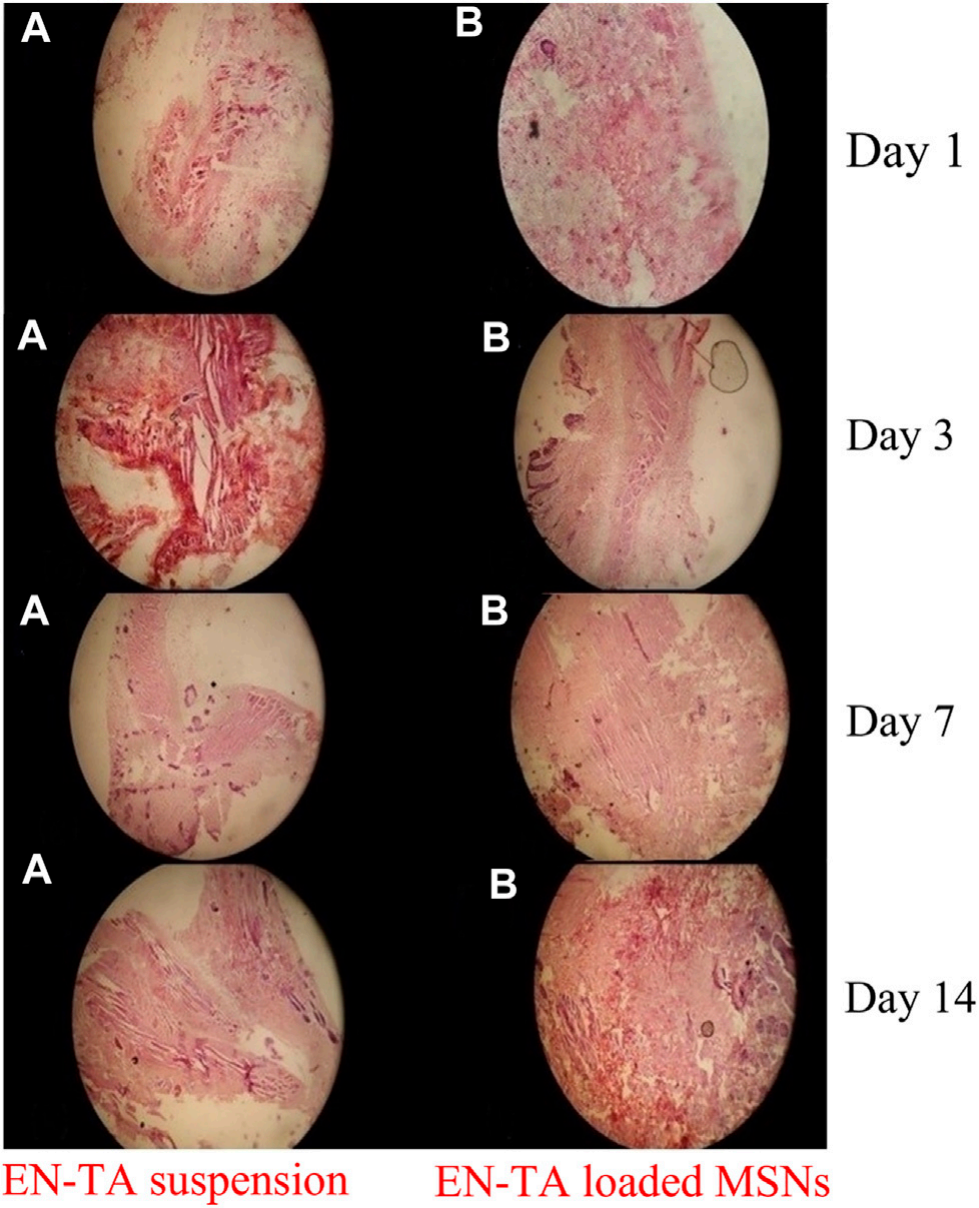
Histopathological changes in pure EN-TA suspension treated control group-I and in EN-TA loaded optimized MSNs treated group-II on day-1, day-3, day-7, and day-14.

On the seventh day, a huge quantity of ECM was still observed in the control group in the form of a leafy structure with less infiltration of immune cells, and the number of fiber strands of wavy form was also very small. The rate of mitosis was comparatively decreased in the EN-TA suspension treated group than in the MSNs treated group. In the MSNs treated group, most of the extracellular fluid was largely infiltrated by macrophages with a slight appearance of follicular bases. Occasionally, the deposition of gas droplets and the rearrangement of T-lymphocytes forming a layer of squamous epithelial cells along with a fast cell division rate were also observed in this treated group. On the 14th day, an ECM-like sheet and some wavy fiber strands were visible in the control group (treated with EN-TA suspension). However, there was a complete lack of presence of follicular cell bases in this group. In the MSNs treated group, most of the extracellular, sheet-shaped structure was replaced by wavy structures like strands, infiltrated by macrophages. The formation of follicular bases and trans-differentiation of cells were detected, and a large number of trans-differentiated cells had appeared, indicating the proliferation and remodeling of skin tissue. Overall, the tissues of the treated group healed quickly, and the skin developed faster than the control group treated with the EN-TA suspension (Figure 8).

## 4 Conclusion

The demand for new systems for the administration of drugs can be successfully satisfied by the development of EN-TA–loaded MSNs. The MSNs were developed by a simple and practical method of sol-gel for prolonged simultaneous administration of antifungal agents for the treatment of the most resistant fungal infections. The optimization of the experimental parameters in order to increase the PY and to have a well-controlled drug release was successfully carried out. Physico-chemical characterizations such as FTIR, DSC/TGA and XRD have shown suitable compatibility of TEOS with both encapsulated drugs along with an indication of some conversion of the crystalline nature of drugs in amorphous forms during MSNs formulation. The uniform distribution of the drugs in the MSNs and the porous structure of the nanoparticles were also evident in SEM studies. In comparison, EN-TA–loaded MSNs exhibited a fast and enhanced healing effect and proved to be less cytotoxic than pure drugs. This improved efficacy of EN-TA–loaded MSNs may be associated with an improvement in the occlusive and bioadhesive properties of MSNs in conjunction with a longer-lasting controlled administration of drugs to the wound site. Itchy skin, rashes, and dry skin associated with conventional methods of use of EN-TA would definitely be reduced by MSNs and will later increase adherence and tolerance in patients. The optimized combination therapy with MSNs has significantly reduced the toxicity of the therapeutic agents by their encapsulation. What is even more interesting is that a synergistic antifungal efficacy was successfully achieved with a reduced dosage of the two active ingredients in combination with MSNs in both *in vitro* and *in vivo* antifungal studies. The promising results of *in vitro* and *in vivo* tests proveD that MSNs have very effective and suitable drug release and wound healing properties for various fungal infections.

## Data Availability

The original contributions presented in the study are included in the article/Supplementary Material, further inquiries can be directed to the corresponding authors.
