# Supplementary material for: Enhanced Antifungal and Wound Healing Efficacy of Statistically Optimized, Physicochemically Evaluated Econazole-Triamcinolone Loaded Silica Nanoparticles
Source: Front Chem. 2022 May 3;10:836678. doi: 10.3389/fchem.2022.836678 (PMC9112326; doi:10.3389/fchem.2022.836678)
Supplement: Supplementary file 1 [file DataSheet1.docx]

**SUPPLEMENTARY DATA**

**Enhanced antifungal and wound healing efficacy of statistically optimized, physicochemically evaluated Econazole-Triamcinolone loaded silica nanoparticles**

Hafeez Ullah Khan^1^*, Hina Younis^1^, Safirah Maheen^1^, Syed Salman shafqat^2^*, Sajed Ali^3^, Atta Ur Rehman^4^, Saliha Ilyas^1^, Muhammad Nadeem Zafar^5^, Syed Rizwan Shafqat^6^, Abul Kalam^7,8^, Ahmed A. Al-Ghamdi^9^

^1^Department of Pharmaceutics, College of Pharmacy, University of Sargodha, Sargodha, Pakistan

^2^Department of Chemistry, Division of Science and Technology, University of Education, Lahore, Pakistan

^3^Department of Biotechnology, University of Management and Technology Sialkot Campus, Sialkot

^4^Department of Pharmacy, Forman Christian College (A Charted University), Lahore 54600, Pakistan

^5^Department of Chemistry, University of Gujrat, Gujrat 50700, Pakistan

^6^Department of Chemistry, Universiti Malaysia Sarwak, Malaysia

^7^Research Center for Advanced Materials Science (RCAMS), King Khalid University, Abha, Saudi Arabia

^8^Department of Chemistry, College of Science, King Khalid University, Saudi Arabia

^9^Department of Physics, Faculty of Science, King Abdulaziz University, Jeddah, Saudi Arabia


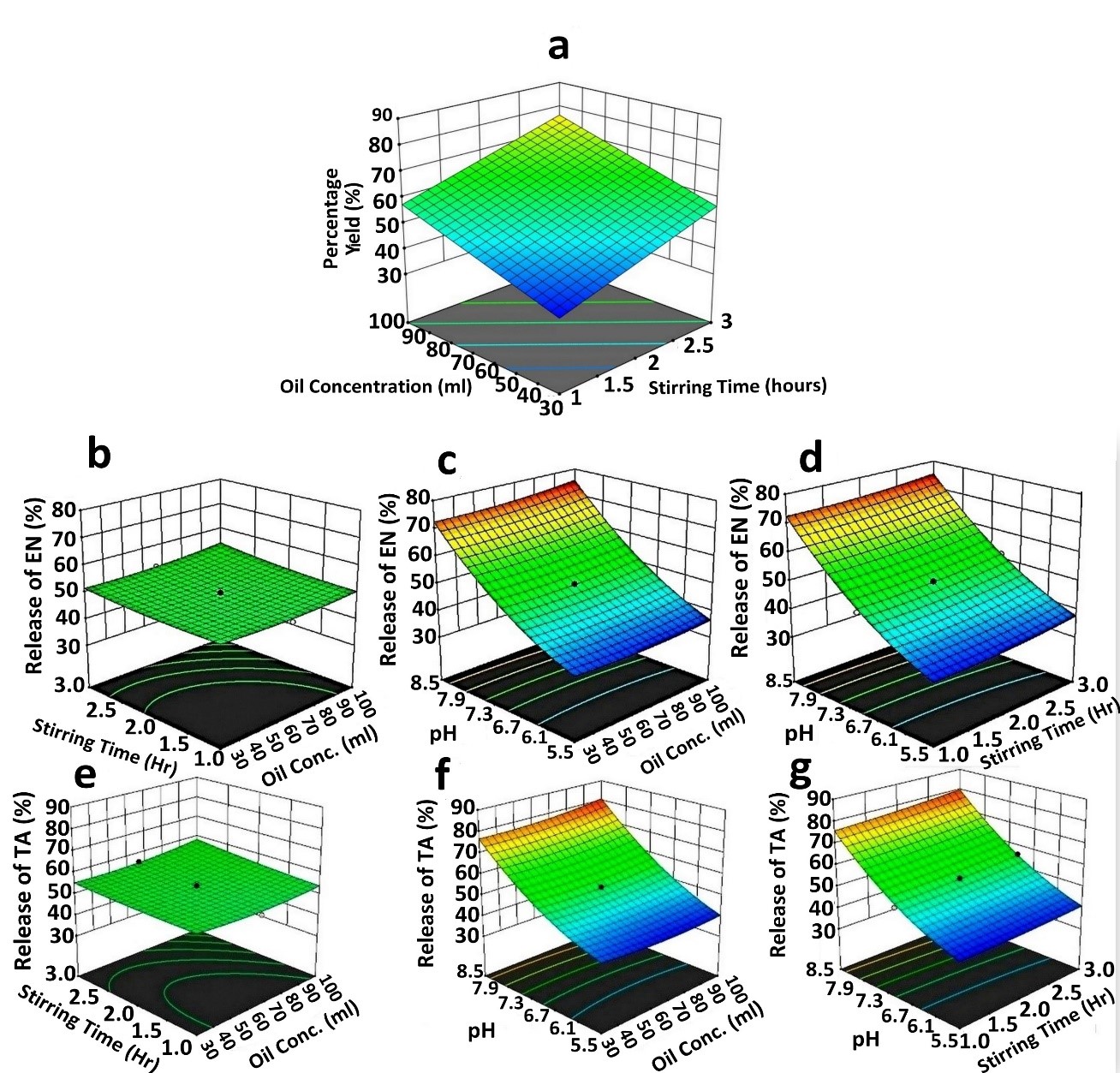


**Figure S1.** 3D plots showing the combination impact of oil concentration, pH and stirring time on Percentage Yield (a), EN release (b, c, d), and TA release (e, f, g)


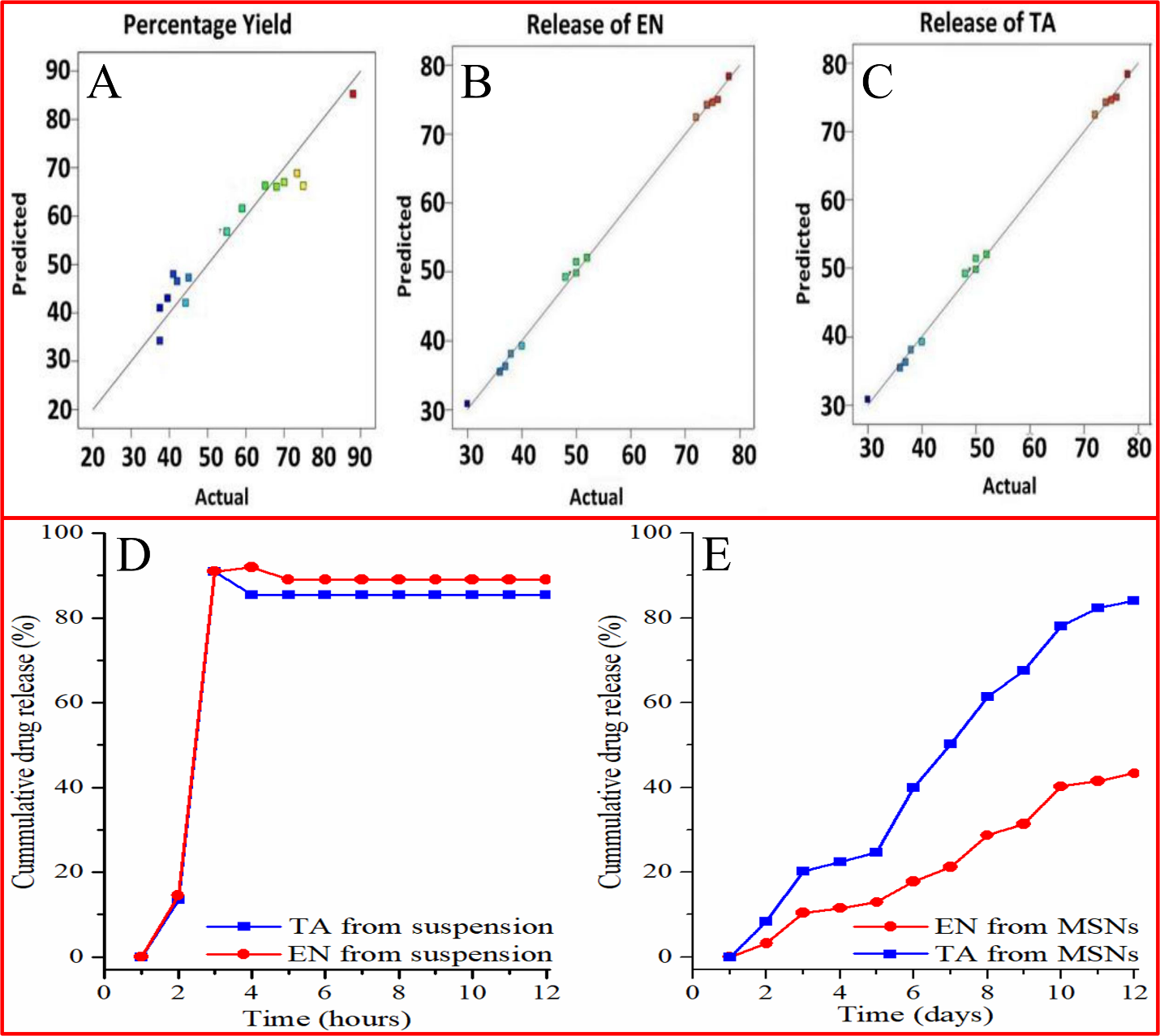


**Figure S2.** Predicted versus actual graphs for percentage yield, EN release, TA release (A-C), *in vitro* release of EN-TA from suspension (D), and *in vitro* release of EN-TA from MSNs (E)

**Table S1.** Different kinetic models with their respective equations to analyze drug release profiles of MSNs

| **Model** | **Mathematical expression of model** |
| --- | --- |
| Zero order model | C=K_o_t  *where C is the drug amount and* K_o_ *is rate constant.* |
| First order model | Log C= Log C_o_ + Kt/2.303  *where Cº is the initial conc and kt is rate constant.* |
| Higuchi model | Q=Kt^1/2^  where *t is time and k is Higuchi rate constant* |
| Hixon-crowell model | C_ο_^1/3^ – C_t_^1/3^ = k_s_t  *where k_s_ is constant for surface volume and C_t_ is remaining amount of drug* |
| Korsmyer-peppas model | Mt/Mα= kt^n^  *where t is time and n is release kinetics constant used to describe the mechanism of drug transport.* |

**Table S2.** Data of different drug release kinetics models for MSNs

| Formulations | Zero order kinetics | | First order kinetics | | Higuchi kinetics | | Hixson-Crowell kinetics | | Korsemeyer-Peppas kinetics | |
| --- | --- | --- | --- | --- | --- | --- | --- | --- | --- | --- |
|  | **R^2^** | **K_0_** | **R^2^** | **K_1_** | **R^2^** | **K_H_** | **R^2^** | **K_HC_** | **R^2^** | **n** |
| F1 | 0.9861 | 19.878 | 0.7259 | 2.672 | 0.8996 | 9.765 | 0.7651 | 1.697 | 0.9548 | 0.970 |
| F2 | 0.9979 | 18.787 | 0.7488 | 2.577 | 0.9475 | 8.943 | 0.5545 | 2.467 | 0.9757 | 1.011 |
| F3 | 0.9991 | 18.466 | 0.6666 | 1.865 | 0.8989 | 11.601 | 0.6551 | 2.998 | 0.9671 | 1.007 |
| F4 | 0.9978 | 18.785 | 0.7147 | 1.543 | 0.9572 | 9.879 | 0.7122 | 3.997 | 0.9855 | 0.917 |
| F5 | 0.9978 | 19.766 | 0.6875 | 1.782 | 0.8898 | 8.593 | 0.5335 | 4.145 | 0.9478 | 1.004 |
| F6 | 0.9899 | 17.988 | 0.8286 | 2.852 | 0.9644 | 9.481 | 0.8029 | 1.792 | 0.9631 | 0.807 |
| F7 | 0.9897 | 18.753 | 0.7687 | 1.461 | 0.9722 | 12.304 | 0.7347 | 4.534 | 0.9549 | 1.041 |
| F8 | 0.9992 | 18.681 | 0.7853 | 1.688 | 0.8995 | 9.732 | 0.8329 | 5.173 | 0.9733 | 0.908 |
| F9 | 0.9869 | 19.879 | 0.8372 | 2.367 | 0.9573 | 8.842 | 0.9948 | 4.919 | 0.9623 | 1.013 |
| F10 | 0.9892 | 18.656 | 0.6881 | 1.298 | 0.9685 | 7.678 | 0.6151 | 3.747 | 0.9679 | 1.011 |
| F11 | 0.9979 | 19.887 | 0.5751 | 1.165 | 0.8877 | 6.638 | 0.7239 | 3.742 | 0.9550 | 0.913 |
| F12 | 0.9988 | 19.674 | 0.8497 | 2.498 | 0.9656 | 8.895 | 0.7053 | 1.828 | 0.9434 | 1.013 |
| F13 | 0.9975 | 19.765 | 0.6884 | 1.359 | 0.8982 | 9.756 | 0.8322 | 5.119 | 0.9326 | 0.909 |
| F14 | 0.9896 | 18.889 | 0.8993 | 2.947 | 0.8869 | 8.323 | 0.6164 | 1.853 | 0.9288 | 0.912 |
| F15 | 0.9987 | 18.772 | 0.7868 | 2.834 | 0.9565 | 7.268 | 0.9266 | 2.754 | 0.9343 | 1.022 |
| F16 | 0.9972 | 19.681 | 0.5887 | 2.723 | 0.9652 | 6.595 | 0.7557 | 4.815 | 0.9453 | 0.892 |
| F17 | 0.9883 | 18.767 | 0.7576 | 1.582 | 0.8733 | 9.427 | 0.6329 | 3.928 | 0.9285 | 1.014 |
| F18 | 0.9895 | 19.864 | 0.8949 | 3.653 | 0.8837 | 8.479 | 0.8458 | 3.923 | 0.8921 | 0.898 |
| F19 | 0.9959 | 18.773 | 0.7968 | 1.542 | 0.8724 | 7.304 | 0.9749 | 2.184 | 0.9134 | 1.021 |
| F20 | 0.9955 | 17.859 | 0.6657 | 2.301 | 0.9905 | 8.638 | 0.7531 | 2.297 | 0.8893 | 0.982 |

Table S3. Micromeritics and entrapment efficiency (EE) of drugs loaded MSNs

| **Formulations** | **Hausner^’^s ratio** | **Carr’s index** | **Angle of repose** | **Triamcinolone**  **EE (%)** | **Econazole**  **EE (%)** |
| --- | --- | --- | --- | --- | --- |
| F1 | 1.11±1.68 | 14±1.59 | 19±2.63 | 79±1.45 | 82±2.83 |
| F2 | 1.08±1.27 | 11±2.54 | 16±3.49 | 58±2.68 | 60±3.72 |
| F3 | 1.14±1.71 | 16±1.87 | 14±1.18 | 73±3.92 | 74±2.96 |
| F4 | 1.09±2.43 | 13±3.43 | 21±2.29 | 53±3.87 | 55±3.51 |
| F5 | 1.07±3.76 | 10±1.56 | 18±2.17 | 68±1.54 | 71±1.89 |
| F6 | 1.10±3.71 | 12±3.42 | 20±1.82 | 59±2.79 | 60±2.62 |
| F7 | 1.06±1.29 | 11±2.23 | 20±2.25 | 62±3.48 | 64±3.29 |
| F8 | 1.09±2.58 | 12±1.29 | 19±3.09 | 60±2.67 | 62±3.87 |
| F9 | 1.07±3.93 | 09±1.69 | 17±2.14 | 59±1.19 | 60±1.98 |
| F10 | 1.15±3.66 | 17±2.78 | 19±3.03 | 56±3.29 | 59±3.95 |
| F11 | 1.18±1.77 | 19±3.76 | 17±2.71 | 40±3.67 | 42±2.23 |
| F12 | 1.19±2.52 | 20±1.34 | 14±1.96 | 73±2.31 | 77±2.76 |
| F13 | 1.18±1.79 | 19±2.13 | 22±2.76 | 37±2.46 | 40±3.47 |
| F14 | 1.12±2.42 | 12±1.44 | 19±3.15 | 39±3.28 | 41±3.58 |
| F15 | 1.15±1.93 | 10±3.31 | 19±1.49 | 71±3.98 | 76±2.78 |
| F16 | 1.19±1.56 | 16±1.58 | 22±2.31 | 54±2.94 | 59±2.63 |
| F17 | 1.16±2.13 | 12±3.28 | 18±3.11 | 35±2.27 | 38±2.35 |
| F18 | 1.18±2.43 | 19±2.91 | 21±2.73 | 36±1.71 | 40±2.16 |
| F19 | 1.21±1.51 | 20±3.25 | 20±1.97 | 59±2.83 | 62±1.96 |
| F20 | 1.19±2.32 | 18±1.98 | 21±3.08 | 72±3.39 | 78±2.79 |
